# Regulatory Mode and Risk-Taking: The Mediating Role of Anticipated Regret

**DOI:** 10.1371/journal.pone.0143147

**Published:** 2015-11-18

**Authors:** Angelo Panno, Marco Lauriola, Antonio Pierro

**Affiliations:** 1 Department of Education, Experimental Psychology Laboratory, Roma Tre University, Rome, Italy; 2 Department of Social & Developmental Psychology, University of Rome “Sapienza”, Rome, Italy; University of Pennsylvania, UNITED STATES

## Abstract

We propose that decision maker’s regulatory mode affects risk-taking through anticipated regret. In the Study 1 either a locomotion or an assessment orientation were experimentally induced, and in the Studies 2 and 3 these different orientations were assessed as chronic individual differences. To assess risk-taking we used two behavioral measures of risk: BART and hot-CCT. The results show that experimentally induced assessment orientation–compared to locomotion–leads to decreased risk-taking through increased anticipated regret (Study 1). People chronically predisposed to be in the assessment state take less risk through increased anticipated regret (Study 2 and Study 3). Study 2 results also show a marginally non-significant indirect effect of chronic locomotion mode on BART through anticipated regret. Differently, Study 3 shows that people chronically predisposed to be in the locomotion state take greater risk through decreased anticipated regret, when play a dynamic risk task triggering stronger emotional arousal. Through all three studies, the average effect size for the relationship of assessment with anticipated regret was in the moderate-large range, whereas for risk-taking was in the moderate range. The average effect size for the relationship of locomotion with anticipated regret was in the moderate range, whereas for risk-taking was in the small-moderate range. These results increase our understanding of human behavior under conditions of risk obtaining novel insights into regulatory mode theory and decision science.

## Introduction

Although some people take more risks than others, almost any human endeavor carries some element of risk. Broadly speaking, people take different types of risk in making everyday decisions as far as there is potential for a choice to result in an undesirable outcome (i.e., a loss). Some authors [1, 2] have suggested that people tend to appraise risk in terms of possible negative outcomes, rather than chance probabilities as posited by classic economic theory (i.e., [3]). In keeping with this view, some works (e.g., [4, 5, 6]) have shown that the risk dimension encompasses a 'strong fear' of losses, characterized by lack of control. Based on these studies, decision makers’ self-regulatory orientation may play a relevant role in understanding human behavior under conditions of risk. Indeed, a specific self-regulatory competence has been shown to influence the degree of risk-taking propensity [7]. Self-regulation can generally be viewed as the ability to control, modify, and adapt one’s behavior in consideration of one’s own emotions [8].

Several authors provided evidence that people often take risk based on anticipated emotions triggered during decision-making processes [4, 9, 10, 11, 12, 13]. According to Loewenstein and colleagues, anticipated emotions are “emotions that are expected to occur when outcomes are experienced” (p. 269 [4]); for example, decision makers are assumed to anticipate how they will feel about obtaining different outcomes as the result of various counterfactual comparisons. A number of decision making theories also posit a prominent role for anticipated emotions, especially in terms of anticipated regret which might arise from prefactual comparisons before making a decision (e.g., [10, 11]). Less attention has been paid to the relationship between decision maker's self-regulatory orientation and anticipated emotion such as anticipated regret, which occurs when risky options are offered. The main purpose of the present research is to examine how decision-maker's regulatory mode (i.e., self-regulatory orientation) affects anticipated regret before taking risky choices. Before presenting data in support of our hypotheses, in the following sections we review the literature providing the theoretical ground for our research.

### Regulatory Mode and Decision Making

A number of researchers have disclosed many factors, which might improve or impair the effectiveness of self-regulation strategies during goal pursuit (e.g., [8, 14, 15]). Compelling results have shown that self-regulatory strategies interact with the emotional stimuli when people need to exert self-regulation [16]. Mischel et al. attempted to shed light on these differences in terms of underlying mediating processes, which involve self-regulatory strategies, emotions, and goals. These authors claimed that a challenge for future research includes a better understanding of how possible mediating individual or context variables interact and guide people's behavior [16]. In this framework, Kruglanski and colleagues have proposed the regulatory mode theory in which two independent goal-oriented motivational factors, like the so-called assessment and locomotion tendencies, are posited to influence people’s behavior, either as chronic personality dispositions or momentarily as situationally induced states. Specifically, the assessment mode “constitutes the comparative aspect of self-regulation concerned with critically evaluating entities or states, such as goals or means in relation to alternatives in order to judge relative quality” (p. 794 [15]). By contrast, the locomotion mode “is the self-regulatory aspect concerned with movement from state to state and with committing the psychological resources that will initiate and maintain goal-directed progress in a straightforward manner, without undue distractions or delays” (p. 794 [15]). In the assessment mode, individuals emphasize critical evaluations (e.g., ‘Which alternative is the best?’). In other words, assessors are concerned with determining the rate, amount, size, or value of something, with critical evaluation guiding action. By contrast, locomotors are concerned with initiating and maintaining movement strongly leading to one’s goal.

Regulatory mode has been included among the most prominent individual difference variables, which may potentially account for choice behavior in decision making [17]; but only recently Panno and colleagues pointed out a correlational relationship between regulatory mode and risk-taking [18]. This study highlighted a negative association between individual differences in assessment mode and risk-taking. To the best of our knowledge, there is no empirical evidence showing an effect of experimentally induced regulatory mode on risk-taking. Furthermore, no studies have shown underlying emotional mechanisms of this relationship.

Previous research pointed out that people who are in locomotion mode are supposed to be dynamic and active decision-makers, whereas people who are in assessment mode are supposed to be concerned with the evaluative aspects of choice options [15, 19]. Camerer, Loewenstein, and Prelec [20] have assimilated the distinction between assessment and locomotion orientation to the distinction between controlled and automatic processes. Consistently with this view, Mannetti and colleagues pointed out that if the locomotion mode overrides the assessment mode, more impulsive choices and decisions would be made. By contrast, if the assessment mode overrides the locomotion one, less impulsive, systematic, and more far-sighted choices will result [21]. For example, locomotion is related to willingness to make prompt decisions, to quickly initiate actions, and maintain them without disruption. By contrast, assessment is related to evaluation of each option before making decisions in order to deeply investigate and appraise the alternatives [15, 22].

Avnet and Higgins demonstrated that a direct and causal link exists between regulatory mode and decision-making processes as they demonstrated that experimentally induced assessment and locomotion orientations affected people's purchasing behavior [23]. Such study provides a powerful research design: by manipulating regulatory mode processes directly, it can demonstrate causal effects of particular strategies on dependent variables of interest. Consistently, a recent review highlighted that regulatory mode of self-regulation affects basic processes of judgment, including the evaluation of alternative hypotheses or counterfactuals, thus underpinning the relationship between regulatory mode and decision-making processes [24]. Pierro and colleagues provided empirical evidence that regulatory mode—both experimentally induced or as personality dispositions—affected the experience of post-decisional regret [25]. The theoretical account for these results is related to a greater amount of counterfactual thinking, which in turn is related to more experience of regret. The latter is supposed to be stronger for people in assessment mode based on their aptitude to make effortful and full comparisons in decision making; whereas people in locomotion mode are supposed to experience less post-decisional regret based on their tendency to move suddenly from state to state, thereby leaving less room for counterfactual thinking [25]. Existing research has related the regulatory mode to processes underlying people's decision making [23, 25]. Nevertheless, this literature mostly considered consumer behavior outcomes. In addition, although post-decisional regret has been thought to be a consequence of regulatory mode (e.g., [25]), less is known about whether anticipated regret can be considered a mediator of the relationship between regulatory mode and risk-taking. Since Mellers and colleagues [11] pointed out that anticipated emotions related to desired and undesired future outcomes are crucial in making risky decisions, then one may consider anticipated regret as the most prominent mediator of the relationship between regulatory mode and risk-taking. Keeping this view in mind, we believe not only that the effect of regulatory mode on risk can be detected, but also that anticipated regret is a key element in explaining mechanisms underlying this relationship. On the one hand, these hypotheses are based on Pierro et al.'s results [25], which showed that regulatory mode affected the post-decisional regret. On the other hand, they are based on the results of decision-making research, which showed that the anticipation of negative emotional reactions (e.g., regret) affects risk-taking [11]. Combining regulatory mode research [15] with decision science (e.g., [11, 26, 27, 28]), we endeavored to shed light on how anticipated regret stems from decision maker's regulatory mode, and in turn affects risk-taking.

### Anticipated Regret and Risk-Taking

Before presenting the specific goals of this study, let us now provide a more detailed description of the relationship between anticipated regret and risk-taking. Research on regret (e.g., [29, 30, 31, 32, 33, 34, 35]) covers a wide range of life domains (e.g., mental health outcomes, risky decision making, interpersonal relationships), and it is one of the most common emotions studied [36]. Therefore, to understand the trajectory of regret—how regret forms and influences behavior—is indeed crucial across different fields (i.e., psychology, economics, medicine, marketing, neuroscience and so forth).

Anticipated regret can be defined as a cognitively based anticipated emotion that people trigger when considering whether future outcomes would have been better if a different decision had been made. The idea is that this emotion is anticipated and taken into account when people are evaluating different risky options [5, 10, 11, 35]. For example, before making a decision, one can anticipate regret whether he/she thinks the decision will go awry. The expected feedback on the decision's potential outcome then triggers prefactual thinking, protecting against the possibility of experiencing severe regret about not choosing the better decision. The rationale behind the working of anticipated regret is that when future regret is brought to the attention of the decision maker (i.e., just before the decision is made), this feeling will bear weight in the decisional process [11, 29, 31]. In other words, people consider the possibility of future regret before making their decisions [10]. It is worth noting that, one’s anticipated regret is made more salient when risky options are offered as elements of uncertainty and the potential for losses strongly trigger this emotion (e.g., [4, 5, 11]).

Classical regret studies have shown that anticipated regret is related to decision making under conditions of risk (e.g., [26, 27, 28, 30, 32, 33, 34, 35, 37, 38, 39, 40, 41]). A number of studies have demonstrated that anticipated regret increases one’s perception of risk (e.g., [26]) and that it is also strongly related to decreased risk-taking in health behaviors, such as unprotected sex (e.g., [27, 28]), binge-drinking behavior (e.g., [32]), gambling (e.g., [33, 34]), and adolescent smoking (e.g., [30]). Other studies also showed that anticipated regret might lead to riskier choices in some specific contexts such as lottery [35, 38] and negotiation [40].

It is noteworthy that during the past decade or so, behavioral decision scientists have moved from static lotteries and decision scenarios (e.g., [39]) to dynamic risk measures (e.g., [42, 43]), which engender a naturalistic metaphor triggering a relatively strong affective response (i.e., a sense of escalating tension) that mimics, in a controlled environment, the affective phenomenological experience of risk-taking typical of naturalistic environments [2]. In addition, such dynamic risk tasks involve feedback-related emotional processes triggered by the processing of outcomes following participants’ decisions. These features reinforced our decision to study the mediating role of anticipated regret through two of the most popular behavioral measures in this specific class of tasks: i.e., Balloon Analogue Risk Task (BART [44]) and Columbia Card Task (hot CCT [42, 43]). To the best of our knowledge, none of the previous studies have investigated the effect of anticipated regret on risk-taking using a task such as the BART or CCT.

## Hypotheses and Plan of Study

On the one hand, social psychologists have shown that the regulatory mode affects post-decisional regret [25]. On the other hand, the empirical evidence–in decision research–has strongly shown a relationship between anticipated regret and risk (e.g., [11, 26, 27, 28, 33, 39]). Thus, combining the regulatory mode research with decision science, we propose that decision maker’s regulatory mode–either as chronic individual differences or as an experimentally induced state–is related to risk-taking through anticipated regret. Specifically, we hypothesized that people with a stronger assessment orientation will have a stronger propensity to trigger anticipated regret because of their concern with critical evaluation and their tendency to make comparisons. Kruglanski and colleagues pointed out that assessors are concerned with ‘doing the right thing’ [15]. Since a negative outcome implies having failed ‘‘to do the right thing,” people with a strong assessment orientation should be more prone to trigger feelings of anticipated regret, and consequently, would show a less risk-taking tendency. By contrast, we hypothesized that people with a stronger locomotion orientation will have a weaker propensity to trigger anticipated regret because of their concern with moving smoothly from state to state, without hesitation or interruption. Thus, people with a strong locomotion orientation are less likely to pay attention to safer choices, and consequently, would make riskier choices.

We carried out a comprehensive series of three studies in order to investigate the expected relationships. In the first study, we tested the effect of experimentally induced regulatory mode on risk-taking through anticipated regret. Once this relationship was established, we devised the second study to demonstrate the effect of chronic regulatory mode predispositions on risk-taking through anticipated regret. Finally, previous research has shown that chronic locomotion predisposition could be unrelated to BART because “locomotors approach this risk task by playing the game for its own sake rather than setting a strategic plan” (p. 213 [18]). In addition, some authors (e.g., p. 68 [13]) claimed that participants could misconstrue the BART as stationary because it triggers less emotional arousal than other risk tasks such as the hot CCT. Then, we devised the Study 3 with two relevant characteristics addressing this issue. First, we used a dynamic risk task specifically designed to trigger emotional arousal (i.e., hot CCT [43]). Second, we used two items of anticipated regret which did not focus on the anticipated regret that participants felt before taking the task but they measured general anticipated regret that people feel during daily experiences.

## Study 1

### Participants

Participants were 110 undergraduate students (75% women; M_age_ = 25.37, SD = 3.95). All research participants were informed that the top 10 participants, sorted in descending order by their total earnings on the BART, would be rewarded with a prepaid mobile phone card. All participants further received course credit for their participation. In the present and following experiment, we determined the sample size based on a previous meta-analysis, which investigated the people's performance in taking risk using the BART [45].

### Ethics Statement

All participants provided oral informed consent to participate in all of these experiments, and permission to conduct the experiments as well as consent procedure were approved from the Ethics Committee of the University of Rome “Sapienza”. Participants provided oral informed consent after reading a form. A written consent was not asked as we wanted to guarantee the anonymity of our participants who were also our students.

## Procedure

Participants were randomly assigned to either an assessment (N = 56), or a locomotion (N = 54) condition. To induce participants' locomotion or assessment mode, we asked them to think of three different situations in which they personally exemplified either high locomotion or high assessment behaviors and to write them down [23]. For assessment, they were asked to ‘think of some occasion in which you compared yourself with other people’; ‘think of some occasion in which you thought about your positive and negative characteristics’; ‘think of some occasion in which you critiqued work done by others or yourself’. For locomotion, they were asked to ‘think of a day when you made many different things’; ‘think of a time when you finished one project and did not wait long before you started a new one’; ‘think of a time when you decided to do something and you could not wait to get started’. Previous empirical evidence has shown that this experimental design successful induced assessment or locomotion mode, thus demonstrating the causal effect of a particular strategy on dependent variables of interest (e.g., [21, 23, 25]). As regulatory mode manipulation-check, we used two self-report items derived and adapted from Kruglanski and colleagues [15]–Regulatory Mode Questionnaire–(i.e., ‘I am very self-critical and self-conscious about what I am doing’ and ‘By the time I accomplish a task, I already have the next one in mind’, for assessment and locomotion orientations respectively). Then, we assessed participants’ anticipated regret. Finally, participants played the BART in an individual setting on a desktop computer, according to the procedure described by Lejuez et al. [44].

### Measures

#### Anticipated Regret

Anticipated regret was assessed by asking participants to rate ‘How much regret would you feel if you missed out on the game's prize?’ on a 7-point Likert scale with the response anchored at the ends with 1 (No regret) and 7 (Extreme regret) (for similar procedure, see [26]).

#### Balloon Analogue Risk Task

The BART is a computerized task modeling real-world risk behavior through the conceptual frame of balancing the potential for reward and harm [44]. In the task, the participant is presented with 30 trials in which they are asked to inflate a balloon by clicking a specific button on the screen. For each pump, the balloon inflates and $.05 is accrued in a temporary bank. However, balloons can explode anytime during the task, with an explosion probability of 1/128 on the first pump. The participant can decide whether to collect the money in the temporary bank by transferring it to a permanent bank. If the balloon pops before the participant collects the money, all earnings for that balloon are lost and the next balloon is presented. Thus, each pump confers greater risk, but also greater potential reward. It is worth noting that the participant was not informed about the expected balloon breakpoint.

The primary BART score is the average number of pumps of unexploded balloons, also referred to as average adjusted pumps (AAP), with higher scores indicating greater risk-taking. A number of studies used the average adjusted pump as behavioral criteria [45]. These studies, which compared experimental groups versus control groups, provided evidence that the average adjusted pumps was sensitive to a variety of experimental manipulations (e.g., sleep deprivation, medical therapies, or cravings [46, 47, 48, 49, 50, 51, 52, 53]). Many studies also showed that average adjusted pumps was associated with real-world risk behaviors occurring outside the laboratory, thereby legitimating the BART as a measure of risk [44, 54, 55, 56, 57, 58, 59, 60, 61, 62].

## Results

Two one-way ANOVAs were carried out to test whether assessment and locomotion orientations were effectively induced. Assessment vs. locomotion conditions differed significantly on the assessment manipulation-check score: *F*(1,109) = 9.00, *p* < .01, (M_assessment_ = 4.45, SD_assessment_ = 1.12; M_locomotion_ = 3.74, SD_locomotion_ = 1.33) Cohen’s *d* = .57. These analyses also showed that assessment vs. locomotion conditions differed significantly on locomotion manipulation-check score: *F*(1,109) = 4.90, *p* < .05, (M_assessment_ = 3.75, SD_assessment_ = 1.10; M_locomotion_ = 4.26, SD_locomotion_ = 1.30) Cohen’s *d* = .42. These results demonstrated that participants reported to have used the strategy they were instructed to use.

Two one-way ANOVAs were carried out to investigate the effect of experimentally induced regulatory mode on both risk-taking and anticipated regret. We performed these two one-way ANOVAs with the AAP and anticipated regret as dependent variables respectively. We found a significant effect of locomotion orientation (coded 1)–compared to assessment orientation (coded 2)–on AAP, *F*(1,109) = 6.01, *p* < .05, Cohen’s *d* = .46, indicating that participants in the locomotion condition pumped more (M = 34.17, SD = 15.14) than those in the assessment condition (M = 27.58, SD = 12.98). As expected, we also found a significant effect of locomotion orientation (coded 1)–compared to assessment orientation (coded 2)–on anticipated regret, *F*(1,109) = 5.02, *p* < .05, Cohen’s *d* = .43, indicating that participants in the locomotion condition reported less anticipated regret (M = 4.06, SD = 1.47) than those in the assessment condition (M = 4.73, SD = 1.64).

To better investigate mechanisms underlying the relationship between regulatory mode and risk-taking, we tested a mediation model including anticipated regret as a mediator of this relationship.

**Fig 1:**
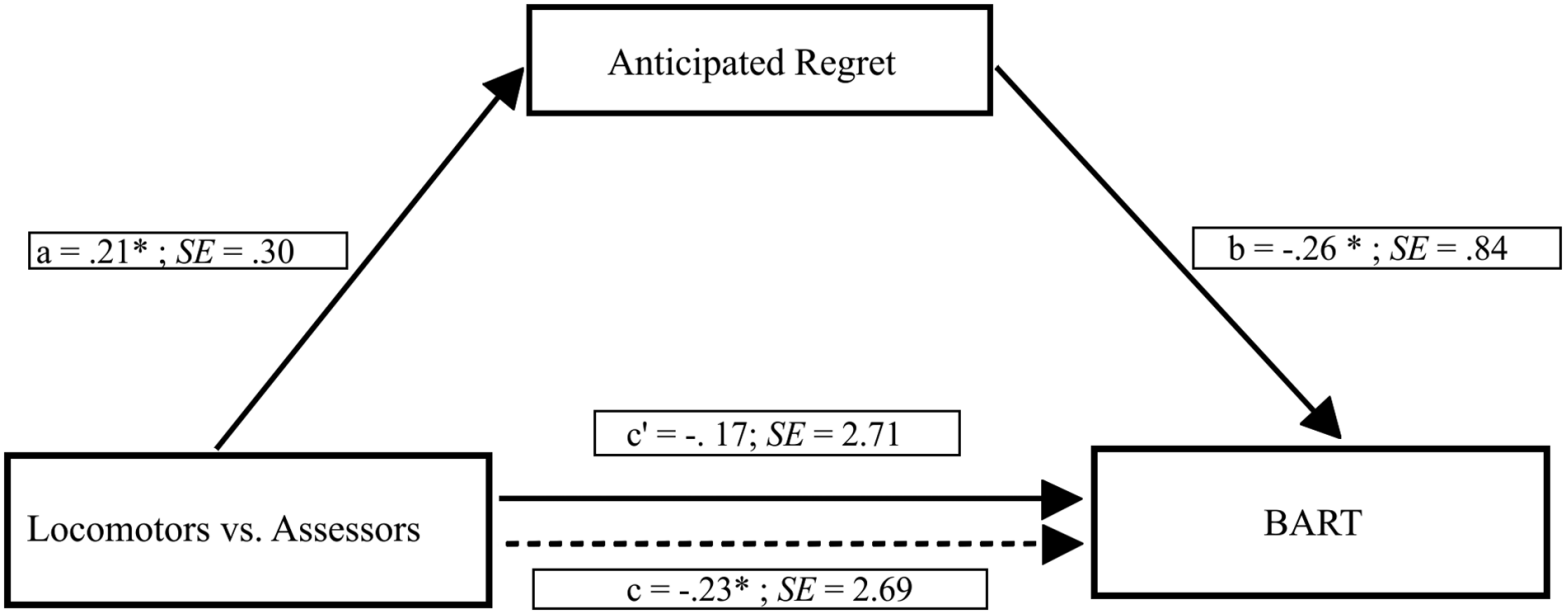
Mediation model showing the effect of experimentally induced locomotion orientation–compared to assessment orientation–on risk-taking through anticipated regret. Path values represent standardized regression coefficients. The (c') value represents the effect, from bootstrapping analyses, of experimentally induced regulatory mode on risk-taking after the mediator is included. Dotted line (c) represents the effect of experimentally induced regulatory mode on risk-taking prior to the inclusion of anticipated regret. *p < .05; **p < .001.

The mediation analysis was performed using the Hayes’s PROCESS macro [63]. A bootstrapping procedure (with 5,000 bootstrap samples) to estimate 95% confidence intervals (95% CI) was used. According to Preacher and Hayes, a 95% CI that does not include zero provides evidence of a significant indirect effect [64]. The bootstrapping procedure has been suggested to represent the most trustworthy test for assessing the effects of mediation models [65]. As shown in Fig 1, the mediation model was estimated to derive the total, direct, and indirect effects of experimentally induced locomotion orientation (coded 1)–compared to assessment orientation (coded 2)–on risk-taking through anticipated regret. We estimated the indirect effect of experimentally induced regulatory mode on risk-taking, quantified as the product of the OLS regression coefficient estimating anticipated regret from experimentally induced regulatory mode (i.e., path *a* in Fig 1), and the OLS regression coefficient estimating risk-taking from anticipated regret controlling for experimentally induced regulatory mode (i.e., path *b* in Fig 1). The mediation analysis provided empirical evidence that a significant negative indirect effect of experimentally induced locomotion orientation–compared to assessment orientation–on risk-taking through anticipated regret was found (point estimate = -1.31, 95% CI = -3.79 to -0.17). Specifically, we found that assessors–compared to locomotors–showed greater anticipated regret, which in turn was related to decreased risk-taking. By contrast, locomotors–compared to assessors–showed less anticipated regret, which in turn was related to increased risk-taking (see Fig 1).

Preacher and Kelley suggested the use of Kappa squared as a measure of effect size for the indirect effect [66]. The Kappa squared is the ratio of the indirect effect to the maximum possible size the indirect effect could have been, given the variances. In the current model, the Kappa squared equaled 0.046 (95% CI = 0.006 to 0.120).

## Study 2

### Participants

Participants were 111 undergraduate students (Mage = 21.28 SD = 3.52; 68% women). As in the previous study, all research participants were informed that the top 10 participants, sorted in descending order by their total earnings on the BART, would be rewarded with a prepaid mobile phone card. All participants further received course credit for their participation.

## Procedure

Participants were tested on two separate sessions, spaced four weeks apart, which were framed as two unrelated studies. In the first session (i.e., testing session), participants filled out the Regulatory Mode Questionnaire (RMQ [15]). The questionnaires were administered in small-group sessions of about eight people. Four weeks later, in the second session (i.e., experimental session), participants played the BART and ratings of anticipated regret were assessed on this occasion.

We chose this experimental design: i) to more conservatively test the predictive power of chronic individual differences in regulatory mode; ii) to reduce suspicion about the goals of the research. This procedure made it more difficult for participants to realize that we were interested in how regulatory mode orientation (measured in the testing session) predicted the extent to which they took risk in the experimental session (for similar procedure, see [67]). The experimenter did not know the participants’ scores on the RMQ (i.e., testing session).

### Measures

#### Regulatory Mode Questionnaire

Kruglanski et al. developed two separate scales, which assessed individual differences in assessment and locomotion orientations [15]. Empirical evidence showed that assessment and locomotion tendencies are relatively independent making it possible for some individuals to score high on both assessment and locomotion, low on both or high on one and low on the other [15]. Kruglanski and colleagues highlighted that locomotion and assessment tendencies are essentially uncorrelated with each other, each contributes to self-regulatory success, and each relates to a distinct task orientation and motivational emphasis. We measured chronic regulatory mode orientation with the RMQ [15], which consists of 24 items using 6-point scales (strongly disagree–strongly agree). Sample items include ‘I often compare myself with other people’ (i.e., assessment mode) and ‘When I finish one project, I often wait awhile before getting started on a new one’ (i.e., locomotion mode, reverse scored). The psychometric properties of the RMQ have been found to be reasonable with alpha reliabilities averaging .80 for locomotion and .73 for assessment [15]. Consistent with these findings, the internal consistencies in our own sample were .72 for locomotion and .78 for assessment. All analyses were based on these continuous measures where higher scores indicated stronger locomotion and assessment orientations.

#### Anticipated Regret

As in the Study 1, anticipated regret was assessed by asking participants to rate ‘How much regret would you feel if you missed out on the game's prize?’ on a 7-point Likert scale with the response anchored at the ends with 1 (No regret) and 7 (Extreme regret) [26].

#### Balloon Analogue Risk Task

As in the previous study (i.e., Study 1), we measured participants’ risk-taking using the BART [44].

## Results

To investigate our hypotheses of the relationships between chronic regulatory mode, anticipated regret, and risk-taking, we computed Pearson correlations among these variables. As shown in Table 1, chronic assessment orientation and anticipated regret were both significantly negatively correlated with AAP. The results of the present study also found that chronic assessment orientation was significantly positively correlated with anticipated regret. All these correlations showed their effect sizes in the moderate range (Fischer’s *Z*_*r*_ ranging between -.25 and .35). The locomotion subscale was not significantly related to either risk-taking or anticipated regret (*p* > .10). The relationship between chronic locomotion orientation and anticipated regret showed a small effect size (Fischer’s *Z*_*r*_ = -.14); whereas, the relationship between chronic locomotion orientation and risk-taking was just around the small effect size threshold (Fischer’s *Z*_*r*_ = .06).

**Table 1:** Means, Standard Deviations and Intercorrelations between Independent Variables, Mediator and Risk-Taking

	1	2	3	4
1 AAP–BART	1			
2 RMQ—Assessment	-.25**	1		
3 RMQ—Locomotion	.06	-.14	1	
4 Anticipated Regret	-.25**	.35**	-.14	1
M (SD)	33.28 (14.93)	3.77 (0.69)	4.20 (0.60)	4.51 (1.53)

**p < .001.

**Fig 2:**
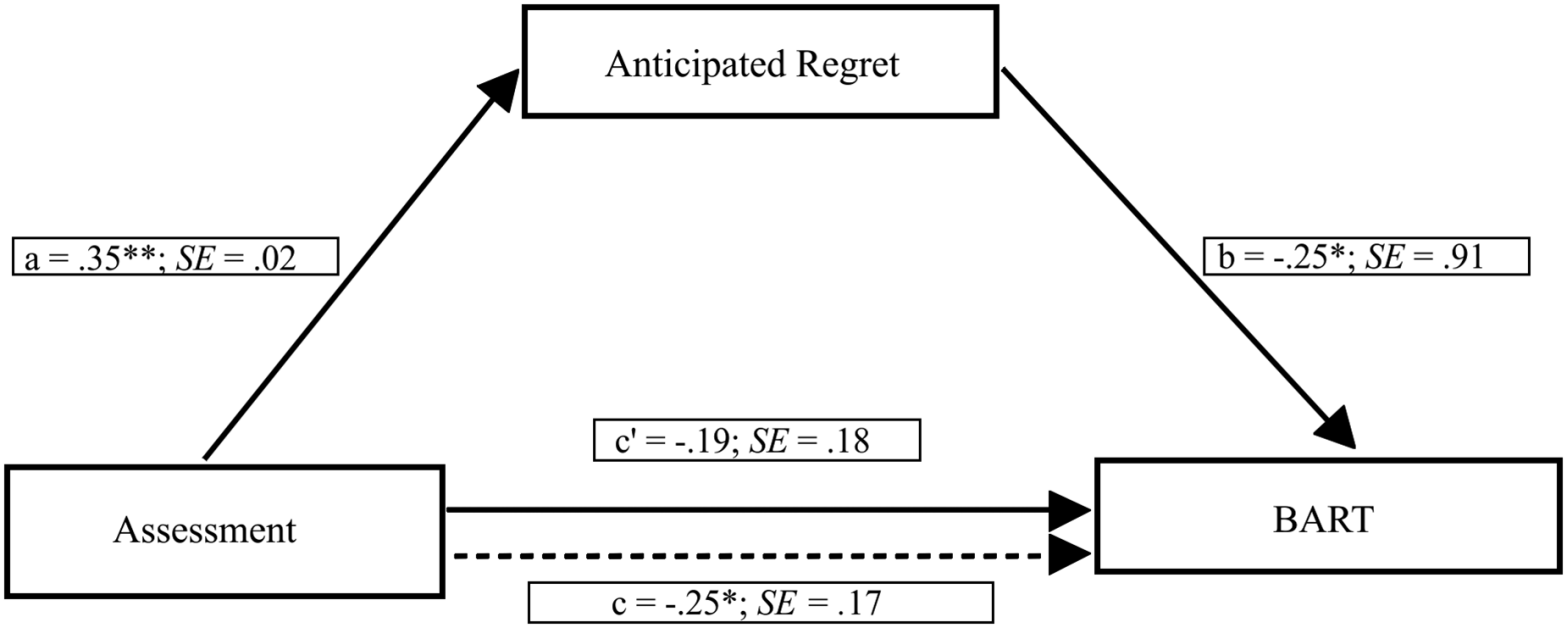
Mediation model showing the effect of the assessment orientation on risk-taking through anticipated regret. Path values represent standardized regression coefficients. The (c') value represents the effect, from bootstrapping analyses, of the assessment orientation on risk-taking after the mediator is included. Dotted line (c) represents the effect of the assessment orientation on risk-taking prior to the inclusion of anticipated regret. *p < .05; **p < .001.

To better understand mechanisms underlying relationships between chronic regulatory mode, anticipated regret and risk-taking, we used the PROCESS macro testing the mediation hypothesis [63]. As shown in Fig 2, we tested a mediation model including chronic assessment orientation as the independent variable, anticipated regret as the mediator and the AAP as the dependent variable. A bias-corrected bootstrap (with 5,000 bootstrap samples) revealed a significant negative indirect effect of the chronic assessment orientation on risk-taking through anticipated regret (point estimate = -0.112, 95% CI = -0.298 to -0.002). Specifically, we found that participants with a stronger assessment orientation showed greater anticipated regret, which in turn was related to decreased risk-taking. In the current model, the Kappa squared equaled 0.060 (95% CI = 0.006 to 0.149).

**Fig 3:**
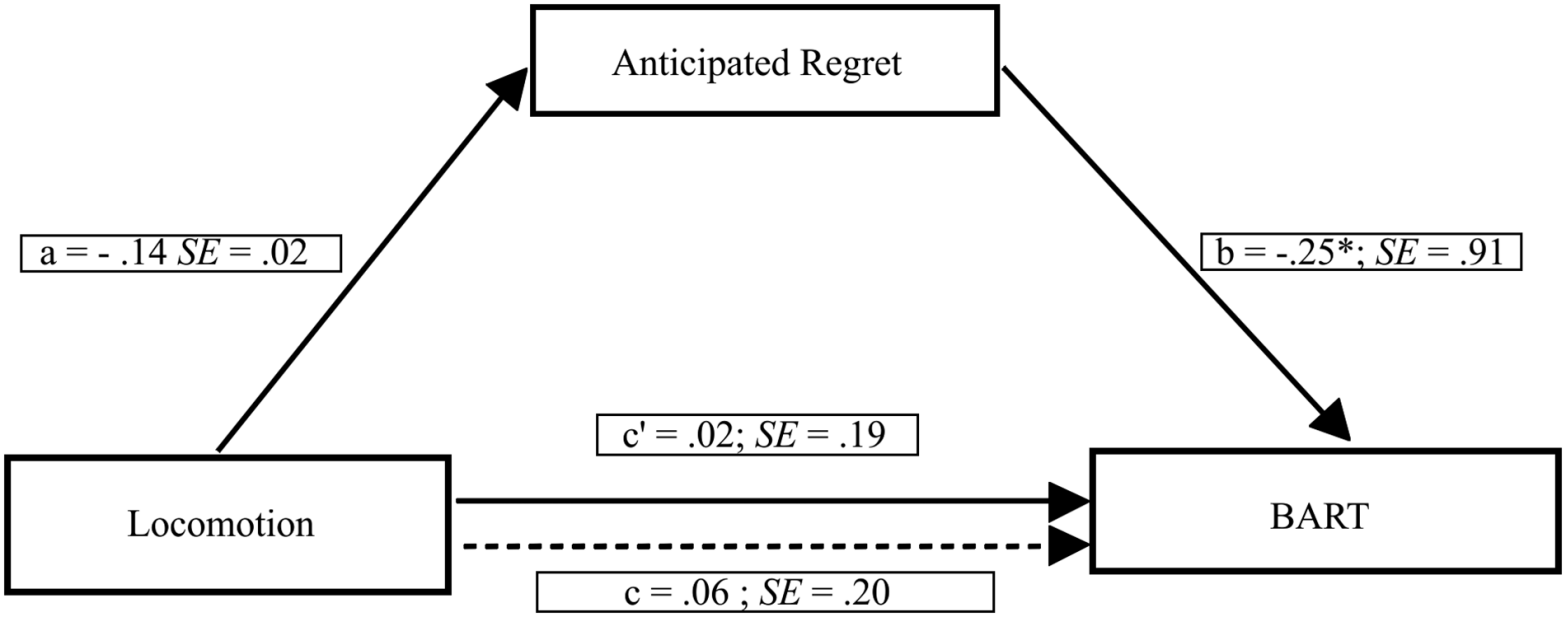
Mediation model showing the effect of the locomotion orientation on risk-taking through anticipated regret. Path values represent standardized regression coefficients. The (c') value represents the effect, from bootstrapping analyses, of the locomotion orientation on risk-taking after the mediator is included. Dotted line (c) represents the effect of the locomotion orientation on risk-taking prior to the inclusion of anticipated regret. *p < .05; **p < .001.

To investigate whether chronic locomotion orientation was related to risk-taking through anticipated regret, we tested a further mediation model including participants’ locomotion orientation as the independent variable, anticipated regret as the mediator and the AAP as the dependent variable (see Fig 3). A bias-corrected bootstrap (with 5,000 bootstrap samples) revealed a marginally non-significant indirect effect of chronic locomotion orientation on risk-taking through anticipated regret (point estimate = 0.068, 95% CI = -0.010 to 0.224). In the current model, the Kappa squared equaled 0.033 (95% CI = 0.003 to 0.101), thus showing a significant small effect size.

## Study 3

### Participants

Participants were 66 undergraduate students (Mage = 21 SD = 2.94; 69% women). They received course credit plus a variable payment in the form of a prepaid mobile phone card whose amount was determined by the outcome of one of the CCT game rounds (with 1 point = 1 cent), randomly selected at the end of the task. In the present study, we determined the sample size based on previous research, which investigated the people's performance in taking risk using the CCT (e.g., [12]).

### Procedure

As in the previous study, participants were tested on two separate sessions, spaced four weeks apart, which were framed as two unrelated studies. In the first session (i.e., testing session), participants filled out the RMQ [15]. During the second session (i.e., experimental session), participants played the hot version of the CCT in an individual setting on a desktop computer, according to the procedure described by Figner and Weber [43]. Participants’ anticipated regret was assessed on this occasion.

### Measures

#### Regulatory Mode Questionnaire

We measured individual differences in regulatory mode with the RMQ [15]. The internal consistencies in our own sample were .78 for locomotion and .70 for assessment. All analyses were based on these continuous measures where higher scores indicated stronger locomotion and assessment orientations.

#### Anticipated Regret

Participants’ anticipated regret was assessed with two, 7-point items (strongly disagree–strongly agree): i.e., ‘Before I act I try to anticipate feelings of regret that would feel whether things go awry’, and ‘Before I make a decision I tend to think a lot about the negative outcomes that might occur’. A composite score of these two items indicated participants’ anticipated regret. Internal consistency for the current sample was .83. All analyses were based on this continuous measure where higher score indicated greater anticipated regret.

#### Columbia Card Task

Participants’ risk-taking was assessed using the hot version of the CCT (hot CCT [43]). It is a 24-trial computer-based measure showing 32 cards face down and a score of 0 points. Participants click on one card after another until they either decide to stop or they turn over a loss card. If participants turn over a loss card, the current trial stops and they lose the stated amount of points. Turning over more cards indicates greater potential reward, but also greater risk. Accordingly, the indicator of risk-taking is the average number of cards chosen per trial with higher scores indicating greater risk-taking [42]. Several experiments using self-reports, skin-conductance measurement, and convergent validity with other measures established that the hot CCT triggers a greater amount of emotional arousal than the cold version of the task [42]. The hot CCT was specifically designed to trigger substantial involvement of affective decision-making processes, as it incorporates an immediate feedback about participants’ choices. Thus, it was demonstrated that the hot CCT triggers dynamic and affective dimensions (e.g., the exhilaration, tension, and regret) that may accompany risky situations [13]. Therefore, this task represents an optimal compromise between inferring risk-taking tendencies from observed choices in a controlled environment and assessing one’s personal involvement in real-life risky situations [43].

## Results

To investigate the relationships between chronic regulatory mode, anticipated regret, and risk-taking, we computed Pearson correlations among these variables. As shown in Table 2, participants’ anticipated regret was significantly negatively related to both chronic locomotion orientation and risk-taking. The results of the present study also found that chronic assessment orientation was significantly positively correlated with anticipated regret. All these correlations showed their effect sizes in the moderate-large range (Fischer’s *Z*_*r*_ ranging between -.26 and .44). Assessment and locomotion subscales were not significantly related to risk-taking (*p* > .10). The relationship between chronic locomotion orientation and risk-taking showed a small-moderate effect size (Fischer’s *Z*_*r*_ = .14). The relationship between chronic assessment orientation and risk-taking was just around the small effect size threshold (Fischer’s *Z*_*r*_ = -.06). In this sample, the two regulatory mode measures were only weakly correlated (see Table 2), consistent with previous studies that have generally found a pattern of weak correlations among these scales (see [15, 25] for more details). According to previous researches (e.g., [15]), in our sample these measures showed only 8% of shared variance, thus supporting their independence.

**Table 2:** Means, Standard Deviations and Intercorrelations between Independent Variables, Mediator and Risk-Taking.

	1	2	3	4
1 Hot CCT	1			
2 RMQ—Assessment	-.06	1		
3 RMQ—Locomotion	.14	-.28*	1	
4 Anticipated Regret	-.26*	.44**	-.39**	1
M (SD)	8.89 (2.48)	3.79 (0.67)	4.35 (0.62)	4.49 (1.46)

*p < .05;

**p < .001.

**Fig 4:**
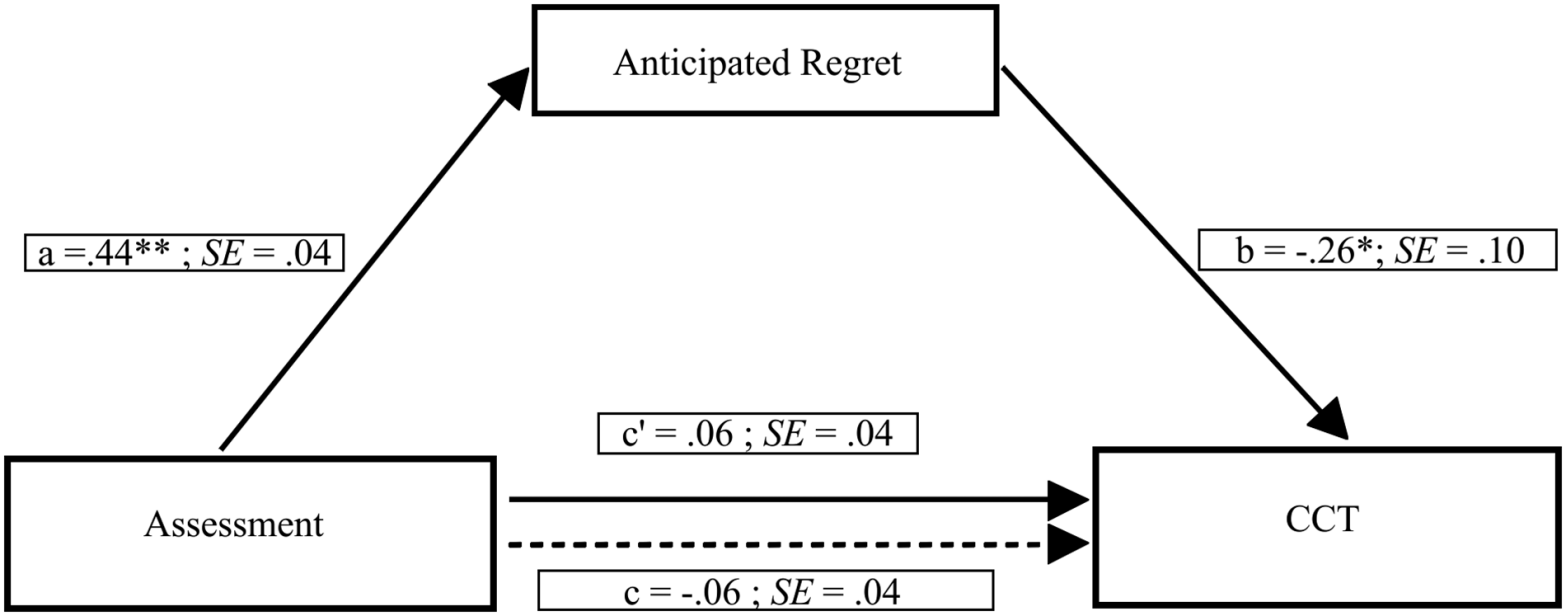
Mediation model showing the effect of the assessment orientation on risk-taking through anticipated regret. Path values represent standardized regression coefficients. The (c') value represents the effect, from bootstrapping analyses, of the assessment orientation on risk-taking after the mediator is included. Dotted line (c) represents the effect of the assessment orientation on risk-taking prior to the inclusion of anticipated regret. *p < .05; **p < .001.

According to Hayes, we proceeded with tests of indirect effects [68]. Indeed, Hayes claims: “researchers not require a significant total effect before proceeding with tests of indirect effects. A failure to test for indirect effects in the absence of a total effect can lead to you miss some potentially interesting, important, or useful mechanisms by which X exerts some kind of effect on Y” (p. 414 [68]). Thus, we used the PROCESS macro to test the indirect effect of regulatory mode on risk-taking through anticipated regret [63]. As in the previous study, we tested a mediation model including chronic assessment orientation as the independent variable, anticipated regret as the mediator and the average number of cards chosen as the dependent variable (see Fig 4). A bias-corrected bootstrap (with 5,000 bootstrap samples) revealed a significant negative indirect effect of assessment orientation on risk-taking through anticipated regret (point estimate = -0.038, 95% CI = -0.091 to -0.006). Specifically, we found that participants with a stronger chronic assessment orientation showed greater anticipated regret, which in turn was related to decreased risk-taking. In the current model, the Kappa squared equaled 0.114 (95% CI = 0.018 to 0.256).

**Fig 5:**
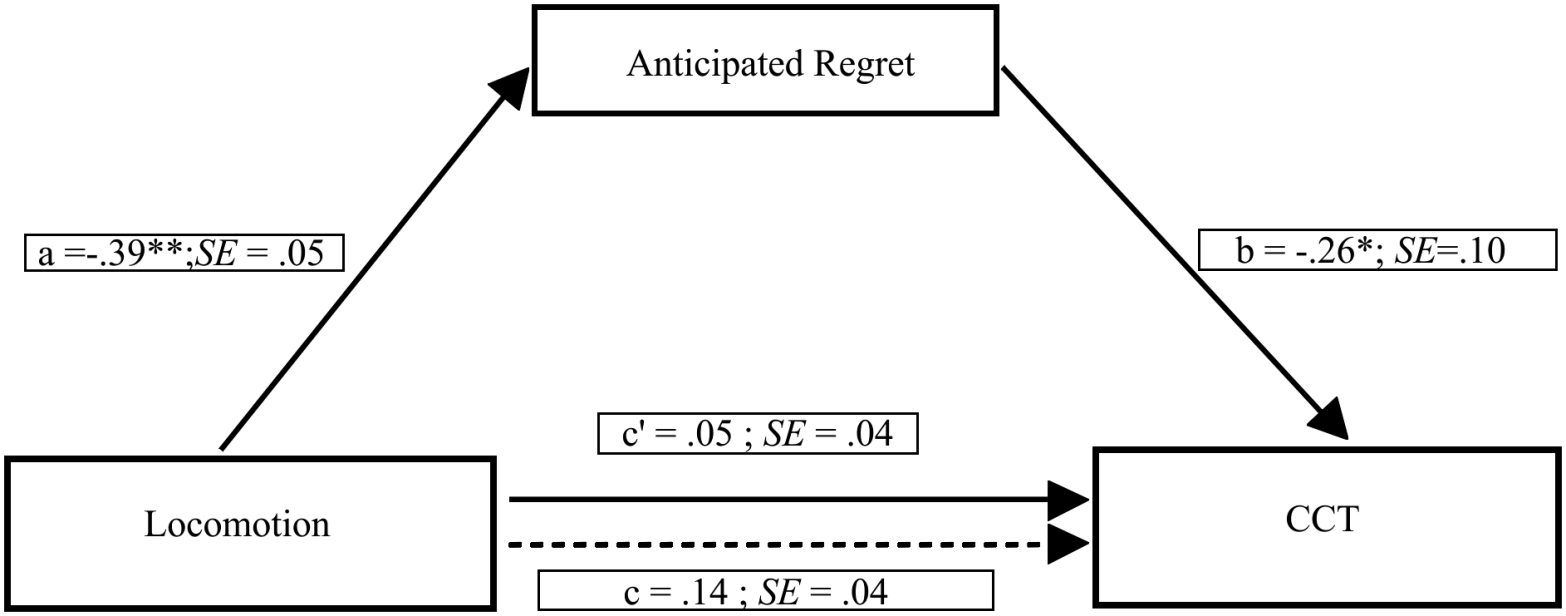
Mediation model showing the effect of the locomotion orientation on risk-taking through anticipated regret. Path values represent standardized regression coefficients. The (c') value represents the effect, from bootstrapping analyses, of the locomotion orientation on risk-taking after the mediator is included. Dotted line (c) represents the effect of the locomotion orientation on risk-taking prior to the inclusion of anticipated regret. *p < .05; **p < .001.

To investigate whether chronic locomotion orientation was related to risk-taking through anticipated regret, we tested a further mediation model including participants’ locomotion orientation as the independent variable, anticipated regret as the mediator and the average number of cards chosen as the dependent variable (see Fig 5). A bias-corrected bootstrap (with 5,000 bootstrap samples) revealed a marginally significant indirect effect of locomotion orientation on risk-taking through anticipated regret (point estimate = 0.030, 95% CI = 0.000 to 0.072). Specifically, we found that participants with a stronger chronic locomotion orientation showed less amount of anticipated regret, which in turn was related to increased risk-taking. In the current model the Kappa squared equaled 0.085 (95% CI = 0.010 to 0.198), thus showing a significant small effect size.

## General Discussion

The results of the current research first provide empirical evidence that experimentally induced regulatory mode affects risk-taking through anticipated regret. Specifically, we demonstrated that the experimentally induced assessment mode–compared to locomotion mode–leads to decreased risk-taking through increased anticipated regret (Study 1). Moreover, we found that people chronically predisposed to be in the assessment state take less risk through increased anticipated regret (Study 2 and Study 3). Consistent with Panno et al. [18], Study 2 results showed a marginally non-significant indirect effect of chronic locomotion mode on BART through anticipated regret. Differently, Study 3 showed that people chronically predisposed to be in the locomotion state take greater risk through decreased anticipated regret, when playing a dynamic risk task triggering stronger emotional arousal.

Based on these results, some distinctions between assessment and locomotion orientations should be made. We believe that two relevant factors can explain the inconsistency between Study 2 and Study 3. First, the measure of anticipated regret, which focused on the anticipated regret that participants felt before to take the task; and consequently, it was not suitable with locomotors’ characteristics because “they approach the BART by playing the game for its own sake rather than setting a strategic plan” (p. 213 [18]). Second, some authors claim that the BART triggers less amount of emotional arousal than other risk-taking tasks (e.g., hot CCT) and, therefore, participants may misconstrue it as stationary [13, 69]. Thus, one might suppose that chronic predisposition of locomotors to engage in exciting activities does not fit with features of a risk task, which does not engender substantial affective dimensions.

To better investigate the link between locomotion orientation and risk taking, we designed the Study 3 focusing on two relevant aspects. First, we used two items of anticipated regret which measured general anticipated regret that people feel during daily experiences. Second, we used a dynamic risk task specifically designed to trigger emotional arousal (i.e., hot CCT [43]). Based on the results of this study, we found a significant indirect effect of chronic locomotion mode on risk-taking through anticipated regret. These results can expand previous research [18] as point out that the indirect effect of chronic locomotion orientation on risk-taking may be detectable when the risk task triggers dynamic and affective dimensions. The current research represents only the first step in this field of investigations, thus further studies are needed to shed light on these mechanisms. Based on our results, we suggest to carefully consider the relationship between locomotion orientation and risk-taking. At the same time, we encourage future studies to adopt different risk tasks that could point out how the relationship between locomotion mode and risk-taking varies across these tasks (see below for more explanations).

Several authors point out the importance to report effect sizes beyond the statistical significance for synthesizing research results and providing two crucial pieces of information: (i) the magnitude of an effect of interest, and (ii) the precision of the estimate of the magnitude of that effect (e.g., [70, 71, 72]). Then, we examine the effects of the two constructs on anticipated regret and risk-taking through a meta-analysis of our studies using the Comprehensive Meta-Analysis Software (CMA, version 2, [73]). Meta-analysis results showed that the average effect size for the relationship of assessment orientation with anticipated regret was in the moderate-large range (Cohen’s *d* = .68, p < .001, 95% CI = 0.38 to 0.98), whereas the average effect size for risk-taking was in the moderate range (Cohen’s *d* = -.41, p < .01, 95% CI = -0.65 to -0.17). For locomotion the average effect size for the relationship of this orientation with anticipated regret was in the moderate range (Cohen’s *d* = -.47, p < .01, 95% CI = -0.77 to -0.18), whereas for risk-taking was in the small-moderate range (Cohen’s *d* = .29, p < .05, 95% CI = 0.05 to 0.53). These results suggest that the effects of assessment and locomotion on anticipated regret were robust. Assessment orientation also showed a robust effect on risk-taking, whereas the effect of locomotion on risk-taking was smaller.

It is noteworthy that, three features make this research well-grounded. First, we demonstrated a causal effect of regulatory mode on risk-taking through anticipated regret via experimental manipulation (Study 1). Second, individual differences in regulatory mode were assessed one month before the experiment in both Study 2 and Study 3. This procedure ensured a conservative test of our hypotheses and obscured to participants that we were interested in how regulatory mode (measured in the first session) predicted the extent to which they took risk in the second session. Third, we assessed risk-taking using the BART as well as hot CCT, which represent a compromise between inferring risk-taking tendencies from observed choices in a controlled environment and assessing one’s personal involvement in real-life risky situations. Thus, the choice to use the BART as well as hot CCT supports the ecological validity of our work.

Although regulatory mode theory has recently been related to risk-taking [18], no study has thus far tested the hypothesis that people’s regulatory mode can influence risk-taking by increasing or decreasing anticipated regret. Relevant to our study, Pierro and colleagues [25] focused on post-decisional regret showing that regulatory mode affects feelings of regret after buying a laptop. Based on our results, we extend Pierro et al.'s research [25] in two ways: First, we show that people's regulatory mode affects feelings of regret even before making a decision. In other words, the decision maker's regulatory mode affects anticipated regret as well as post-decisional regret. Second, we show that experimentally induced assessment and locomotion orientations not only affect consumers’ decision making (e.g., laptop purchase, [25]), but also risk-taking behavior (Study 1). In sum, existing research [15, 23, 25] has shown how regulatory mode is related to different strategies in making decisions. The results of the current studies expand this research showing that people with assessment concerns engage in a greater amount of anticipated regret because they are strategically motivated to make comparisons of all options, when faced with a risky choice [15]. People with locomotion concerns seem to leave little room triggering anticipated regret because strategically motivated to take the action, when faced with a risky choice [15]. With regard to chronic locomotion orientation, we suggest to carefully take these results. Further studies are needed to understand how this relationship can vary across different risk circumstances.

This research has implications for decision-making research into individual differences [17, 18]. The Study 1 results could explain why regulatory mode research yielded unstable findings investigating individual differences in taking risk. Indeed, based on our studies, an experimentally induced regulatory mode could create stability. By contrast, individual differences in regulatory mode could provide instability, as these may be moderated by other variables or by the features of the risk task [17]. Future studies could investigate these relationships using a risk measure assessing risk-taking through different domains such as the DOSPERT. This questionnaire might provide more insights into these connections as it measures people’s risk-taking through health, ethical, gambling, recreational and financial domains [74].

These results could also have important applied implications, as it is not difficult to imagine how people’s regulatory mode could be made more accessible in daily life by situational cues and/or training instructions. For instance, a situationally induced assessment orientation triggering anticipated feelings of regret would increase safer choices during decision-making processes. Moreover, the assessment motive for triggering anticipated regret is of particular interest. In some ways, it relates to self-improvement [15], since “it is about how to make the decision-making process itself better. It involves critical reflection on both what was good and what was bad about the process—the essence of evaluative criticism” (p. 327 [25]). Future studies might then shed light on how this particular ‘critical reflection’ affects further anticipated emotions in making decisions under conditions of risk. Further studies are also needed to explore more fully whether and how the impact of regulatory mode on risk-taking could itself be affected by other personal and situational variables. Although, it was beyond the scope of the current research to investigate all of these, let us consider some possible additional factors of interest. For instance, future work might shed light on how individual differences may mediate or moderate the relationship between decision maker's regulatory mode and risk-taking. Lauriola and Levin [75], for example, found a tendency for neuroticism disposition to have the opposite effect on risk-taking for loss and gain domains. They showed that higher neuroticism scores were associated with lower risk-taking in the gain domain. By contrast, higher neuroticism scores were also associated with greater risk-taking in the loss domain. Moreover, Kruglanski and colleagues have also shown that people's assessment orientation is related to neuroticism disposition [15]. Interestingly, based on these results (i.e., [15, 75]), we may then expect that risk domain could moderate the relationship between the decision maker's assessment orientation and risk-taking. Second, based on previous studies (e.g., [75]), we know that individuals' higher extraversion predicts riskier choices. Regulatory mode research has shown that locomotion mode correlates positively with extraversion (e.g., [15]). Then, future studies should investigate whether people's extraversion can moderate the relationship between locomotion mode and risk-taking. A recent review has shown a link between regulatory mode and the prioritization of fast versus accurate information processing in making judgments [24]. Accordingly, it is easy to imagine how the prioritization of fast versus accurate information processing may affect risk-taking [76]. Thus, the information processing could interact with the effect of the regulatory mode on risk-taking, but future studies are needed to shed light on these mechanisms.

Taken together, the results of the current research increase our understanding of human behavior under conditions of risk obtaining novel insights into decision science, regulatory mode theory, as well as emotion research (e.g., [10, 15, 25]).
